# Obesity trends by industry of employment in the United States, 2004 to 2011

**DOI:** 10.1186/s40608-016-0100-x

**Published:** 2016-04-02

**Authors:** Chandra L. Jackson, Christina C. Wee, David A. Hurtado, Ichiro Kawachi

**Affiliations:** Clinical and Translational Science Center, Harvard Catalyst, Harvard Medical School, Boston, MA USA; Division of General Medicine and Primary Care, Department of Medicine, Beth Israel Deaconess Medical Center, Harvard Medical School, Boston, MA USA; Oregon Institute of Occupational Health Sciences, Oregon Health & Science University, Portland, OR USA; Department of Social and Behavioral Sciences, Harvard T.H. Chan School of Public Health, Boston, MA USA

## Abstract

**Background:**

Obesity is associated with increased morbidity, occupational injuries, and premature mortality. Obesity also disproportionately affects blacks and socioeconomically disadvantaged workers. However, few studies have evaluated national trends of obesity by employment industry overall and especially by race.

**Methods:**

To investigate national trends of obesity by employment industry overall and by race, we estimated the age-standardized obesity prevalence from 2004 to 2011. We used direct age-standardization with the 2000 US Census population as the standard among 136,923 adults in the US National Health Interview Survey. We also estimated prevalence ratios (PRs) for obesity in black women and men compared to their white counterparts for each employment industry using adjusted Poisson regression models with robust variance.

**Results:**

Obesity prevalence increased for men and women over the study period across all employment industry categories, and the healthcare industry had the highest overall age-standardized prevalence (30 %). Black women had a significantly higher obesity prevalence than white women across all employment industry categories, ranging from 33 % (95 % confidence interval (CI): 1.16,1.52) in Professional/Management to 74 % in Education (95 % CI: 1.56,1.93). Obesity prevalence was higher among black than white men for Healthcare (PR = 1.39 [1.15,1.69]), Education (PR = 1.39 [1.17,1.67]), Public Administration (PR = 1.34 [1.20,1.49]), and Manufacturing (PR = 1.19 [1.11,1.27]). Differences in obesity prevalence by race were generally widest in professional/management occupations.

**Conclusions:**

Obesity trends varied substantially overall as well as within and between race-gender groups across employment industries. These findings demonstrate the need for further investigation of racial and sociocultural disparities in the work-obesity relationship to employ strategies designed to address these disparities while improving health among all US workers. Further research and interventions among workers in industries with an increasing or high prevalence of obesity should be prioritized.

**Electronic supplementary material:**

The online version of this article (doi:10.1186/s40608-016-0100-x) contains supplementary material, which is available to authorized users.

## Background

Obesity, a leading public health problem, is associated with an increased risk of multiple chronic conditions including hypertension, type 2 diabetes, cardiovascular disease, certain cancers (e.g. breast), depression and lower quality of life, disability as well as premature mortality [1–4]. US adults who are full-time employees spend approximately one third of their waking hours at work [5], and there is increasing evidence that adverse working conditions can impact obesity risk [6, 7]. Studies suggest adults in certain occupations are more likely to be obese, and obesity—in addition to poor health outcomes—is associated with increased occupational injuries, loss of work-related productivity, and excess healthcare costs [8–10].

Several mechanisms may underlie the occupation-obesity relationship [11, 12]. For instance, obesity may be directly related to occupation through job-related characteristics such as sedentary time (versus physical activity demands), the workplace food environment, work-related stress including job strain, rotating/night shift work, and chemical exposures [13–19]. Individuals working under high-demand, low-control conditions with an effort-reward imbalance are at an increased risk of cardiovascular disease [20], and obesity may be an important contributor. Work demands and pressure likely affect employee eating habits and activity patterns while at work and beyond [3, 21–23]. Additionally, there are indirect effects of occupation on obesity because of income disparities that affect the ability to, for example, afford healthy nutrition [24, 25].

Multifactorial interventions to address the obesity epidemic have been implemented across various settings including the workplace, and some populations have begun to experience plateaus in the prevalence of obesity [26]. Worksite wellness programs represent a potentially promising approach to reduce overall obesity as well as racial/ethnic and socioeconomic disparities by focusing on social, cultural and environmental causes of obesity and not just the individual’s responsibility [27–36]. Some of these interventions appear effective in terms of achieving clinically meaningful reductions in body weight and improved cardiometabolic risk factor profiles through enhanced nutrition and physical activity [28, 29, 32, 33], although not all studies have yielded positive results [27].

Understanding obesity trends across various industries can provide an important opportunity to identify specific industries that could benefit from worksite interventions and reduce obesity risk. Obese, low socioeconomic status adults are over-represented in certain industries. Moreover, such industries may be disproportionately populated by racial/ethnic minorities because of the historical connection between race/ethnicity and occupations. Few studies, however, have examined national trends in obesity by industry to identify the industries with the highest rise in obesity. We also sought to address this gap in the literature by examining obesity prevalence trends across gender and race/ethnicity.

## Methods

### The National Health Interview Survey

We analyzed National Health Interview Survey (NHIS) data, which is a series of nationally representative cross-sectional surveys. Detailed study procedures have been previously published [37]. In brief, the NHIS uses a stratified cluster probability sampling design to conduct in-person interviews among non-institutionalized US civilians. Trained US Census Bureau interviewers collected data among a probability sample of households throughout the year. Using computer-assisted personal interviewing (CAPI), interviewers obtained information about the health status, healthcare services, health-related behaviors, and sociodemographic characteristics of all members of the sampled household. To provide additional health-related information, one adult and one child from each sampled family were randomly selected. The overall response rate for sample adults was 67 % (range: 61–72 %), each study participant provided NHIS with informed consent, and this study was approved by the Harvard T.H. Chan School of Public Health's institutional review board.

### Study participants

Participants included adults who were ≥18 years old and were self-identified Non-Hispanic white or Non-Hispanic black (hereafter, white and black). We focus on Black-White disparities and do not include Hispanics in this particular study because factors (e.g. immigration, culture, acculturation, assimilation) that affect occupational conditions and obesity likely differ by race/ethnicity. Adding an analysis of health disparities along the dimension of ethnicity deserves separate attention. In US data, ethnicity is considered a separate dimension of social stratification as distinct from race (for example, an individual can self-identify as black Hispanic or white Hispanic). Such inter-sectionality—along with the added complexity of introducing variables associated with immigration and acculturation/assimilation—were considered beyond the scope of this present study. Nevertheless, we acknowledge the importance of future studies investigating potential ethnic differences in obesity trends by employment industry among men and women.

Participants were excluded if they 1) were non-US born; 2) had a body-mass index (BMI) considered extreme – <15 or >70 kg/m^2^; 3) were unemployed at the time of the survey; or 4) had missing data (3 %) on BMI, employment industry, or employment status. The final analytic sample included 136,923 employed adults with 30 % considered obese.

### Measures

#### Obesity

We calculated body-mass index (BMI) by dividing self-reported weight in kilograms by self-reported height in meters squared. Obesity was defined as BMI ≥30 kg/m^2^, overweight as 25.0–29.9 kg/m^2^, normal weight as 18.5–24.9 kg/m^2^, and underweight as BMI < 18.5 kg/m^2^.

#### Race/ethnicity

Participants were asked, ‘What race or races do you consider yourself to be?” Participants then chose 1 or more of the following options: white, black/African American, American Indian/Alaskan native, Asian and multiple race. Regarding ethnicity, participants were asked if they were Hispanic or non-Hispanic.

#### Employment status

Determined for all adults, employment status during the week prior to the interview was combined as ‘working for pay at a job/business’, ‘working (but not for pay) at a family owned job/business,’ ‘with a job/business but not at work’, ‘unemployed and looking for work’, and ‘not working at a job or business and not looking for work.’ Unemployed participants were not included in this study.

#### Industry of employment

For stable estimates to investigate potential disparities, we categorized the North American Industrial Classification System (NAICS) codes into eight meaningful categories, including: 1) ‘Agriculture, Fishing, Forestry, and Hunting Industries’; ‘Mining Industries’; ‘Utilities Industries’; ‘Construction Industries’; ‘Manufacturing Industries’; and ‘Wholesale Trade Industries’; and ‘Transportation and Warehousing Industries,’ 2) ‘Retail Trade Industries’, 3) ‘Information Industries’; ‘Finance and Insurance Industries’; and ‘Real Estate and Rental and Leasing Industries’, 4) ‘Professional, Scientific, and Technical Services Industries’; ‘Management of Companies and Enterprises Industries’; and ‘Administrative and Support and Waste Management and Remediation,’ 5) ‘Education Services Industries’, 6) ‘Health Care and Social Assistance Industries’, 7) ‘Accommodation and Food Services Industries’ as well as 8) ‘Other Services (except Public Administration) Industries’; ‘Public Administration Industries;’ and ‘Arts, Entertainment, and Recreation Industries.’

#### Occupation

Adults who ever worked, were working (paid or non-paid) during the week prior to the survey, or who were not at work the week before the survey (although they had a job or business) were asked about their occupation, which was categorized based on the Standard Occupational Classification System codes. We combined occupation categories into ‘Professional/management’, ‘Support Services’ and ‘Laborers’ based on type of work. Based on current, longest held, or most recently held job or work situation, class of work/occupation was categorized as either 1) an employee for wages, salary, or commission at a private company, business, or for an individual; 2) an employee for a federal, state, or local government; 3) self-employed (business, professional practice or farm); or 4) working without pay in a family-owned business or farm.

#### Other socioeconomic factors

Educational attainment was classified as either less than high school (no high school diploma), high school (high school or general equivalency diploma), some college, and a college education or greater. Household annual income was stratified at above and below $35,000. Poverty status was determined as being below the poverty line after considering total income from all sources before taxes among all members of the family.

#### Health behaviors

Lifetime alcohol consumption and smoking status were both categorized as ‘never,’ ‘current,’ or ‘former.’ Physical activity during leisure time was classified as ‘none’, ‘low’, or ‘high’; participants engaging in some level of physical activity and providing a particular number of activity bouts were stratified at the midpoint and categorized as ‘low’ or ‘high.’ Participants reporting ‘never’ or ‘unable to do this type activity’ were categorized as ‘none.’ Sample adults reported how many hours of sleep they, on average, get in a 24-h period, which was categorized as <7 h, 7 to 9 h, and >9 h.

#### Covariates

Age was categorized as 18–49 years, 50–64 years, as well as ≥65 years. Marital status was classified as never married, married/living with partner, or divorced/separated/widowed. General health status (based on self report) was characterized as excellent/very good, good, or fair/poor. US geographic regions were considered as the South, Midwest, Northeast, and West.

### Statistical analysis

We pooled eight survey years (2004–2011) of NHIS data using the Integrated Health Interview Series [38]. Sampling weights were used for all analyses to account for the unequal probabilities of selection to participate in the study, non-response bias, and oversampling of specific subgroups (e.g. racial/ethnic minorities; elderly individuals 65+ years of age). Standard errors were calculated using Taylor series linearization [39]. STATA statistical software version 12 (STATA Corporation, College Station, Texas, USA, 2007) was used for these analyses [40].

Continuous variables were presented as means ± standard errors (SE), and categorical variables were illustrated as absolute values with percentages. To take survey weights into account, Rao-Scott second-order corrected Pearson statistics were employed to test for differences in pre-specified sociodemographic, clinical, and behavioral characteristics overall and between blacks and whites as well as obesity status [41].

To estimate prevalence ratios for obesity overall and among black men and women separately compared to obesity in their white counterparts by employment industry category and occupation with corresponding 95 % confidence intervals, we used 4 different Poisson regression models with robust variance estimation [42]. Pre-specified demographic, health behavior, and occupational characteristics were introduced into the model as a group in a sequential manner. Whites were the reference category for all Black-White comparisons because whites were the largest racial group in this sample, thereby providing greater statistical stability. For models stratified by race and a separate model including an interaction term for race and obesity, we adjusted for age in 3 categories: 18–49, 50–64, 65+ years in the first model, then for sociodemographic factors (e.g. gender, marital status, educational attainment, household income) in the second model. We then adjusted for class of occupation and occupation. We subsequently adjusted for health behaviors, including alcohol consumption, smoking status, leisure-time physical activity and sleep duration to explore the extent to which obesity prevalence may be explained by these lifestyle factors. We also adjusted for prevalent medical conditions (i.e. hypertension, diabetes, cancer, heart disease).

Testing for temporal trends in BMI over time by race and employment industry category, we repeated our analyses stratified by industry and then separately by blacks and whites. Direct standardization for age using the 2000 US Census as the standard population was employed to obtain age-adjusted obesity prevalence estimates. We included interaction terms for race and survey year with 2 years combined in separate linear regression models to investigate possible Black-White differences in BMI prevalence over the study period. In subsequent analyses, we also investigated temporal trends in employment by race-gender groups.

## Results

### Study population characteristics

Among the 136,923 participants, mean age was 47 years, 50 % were women, 13 % were black, 28 % (30 for whites; 17 for blacks) had at least a college education, and 30 % were obese. Table 1 presents weighted estimates of age-standardized obesity prevalence by study population characteristics (i.e. sociodemographic, health behavior, occupational, clinical) among black and white participants. Black women (46 vs. 27 %) and black men (35 vs. 29 %) were more likely to be obese than their white counterparts.

**Table 1:** Age-standardized prevalence of obesity by demographic, occupational, health behavior and clinical characteristics among 136,923 US black and white men and women, National Health Interview Survey, 2004–2011

	Overall	Black women	White women	Black men	White men
	% (95 % CI)	% (95 % CI)	% (95 % CI)	% (95 % CI)	% (95 % CI)
Overall sample size	136,923	15,277	58,784	10,023	52,839
Obese	30 (29.3–30.1)	46 (45.3–47.6)	27 (26.3–27.4)	35 (34.0–36.5)	29 (28.7–29.7)
Age group					
18–49	29 (28.8–29.8)	45 (43.7–46.5)	25 (24.6–26.0)	39 (37.5–40.9)	29 (28.1–29.6)
50–64	33 (32.3–33.6)	51 (48.9–53.2)	30 (29.3–31.2)	35 (32.4–37.0)	33 (32.0–33.9)
≥ 65	25 (24.4–25.6)	41 (39.0–43.7)	24 (23.0–24.8)	29 (26.9–31.8)	24 (22.8–24.7)
Educational attainment					
< High school	33 (32.5–33.8)	47 (45.7–49.2)	31 (30.2–32.1)	35 (32.7–37.3)	33 (31.6–33.4)
High school graduate	37 (35.3–37.8)	53 (50.6–55.8)	35 (32.8–37.0)	37 (34.0–40.1)	34 (31.9–35.3)
Some college	32 (31.1–32.4)	46 (44.1–48.0)	29 (28.2–30.0)	36 (33.9–38.4)	32 (30.5–32.5)
≥ College	22 (21.9–23.0)	40 (37.3–42.4)	19 (18.4–20.0)	34 (30.4–37.5)	23 (22.5–24.1)
Marital status					
Married	29 (28.7–29.6)	46 (44.2–47.9)	26 (24.8–26.2)	38 (36.2–40.1)	31 (30.0–31.2)
Divorced/separated/widowed	31 (30.3–31.6)	47 (45.4–49.0)	29 (28.3–30.1)	34 (32.1–36.4)	28 (26.7–28.8)
Never married	31 (29.8–31.8)	46 (43.7–48.5)	33 (31.0–34.4)	33 (29.5–36.0)	25 (23.3–26.2)
Living in poverty	38 (36.4–38.6)	51 (48.7–53.1)	38 (35.9–39.4)	34 (31.2–37.0)	31 (28.7–32.9)
Household income < $35,000	35 (34.0–35.3)	50 (48.2–51.0)	35 (33.6–35.5)	34 (32.2–35.4)	30 (28.7–30.8)
Class of worker					
Private wage	30 (29.6–30.5)	46 (45.1–47.8)	28 (27.0–28.3)	34 (32.9–35.8)	29 (28.8–30.1)
Government	30 (29.8–31.1)	47 (45.0–49.2)	26 (25.0–26.9)	39 (36.1–41.2)	31 (29.5–32.0)
Self employed	26 (24.7–26.6)	41 (35.5–46.0)	22 (20.7–23.5)	34 (29.0–39.2)	27 (25.2–27.8)
Occupation					
Professional/management	26 (25.5–26.9)	42 (38.8–45.3)	24 (22.5–24.5)	35 (31.8–38.6)	27 (25.6–27.5)
Support services	28 (27.9–28.9)	46 (44.9–47.7)	26 (25.5–26.8)	34 (31.5–36.8)	27 (26.1–28.2)
Laborers	34 (33.2–34.3)	49 (46.6–50.7)	33 (31.6–33.9)	36 (34.4–37.2)	32 (31.4–32.8)
Industry					
Manufacturing/construction	31(30.5–31.6)	47 (44.0–49.6)	28 (26.6–28.9)	35 (33.4–36.9)	31 (30.1–31.6)
Retail	30 (28.6–30.6)	44 (39.7–47.5)	28 (26.4–29.3)	33 (27.8–38.2)	29 (27.7–31.0)
Finances/information	27 (26.0–28.1)	42 (38.0–46.1)	26 (24.9–28.1)	32 (26.7–37.7)	25 (23.6–26.7)
Profess/admin/man	27 (26.0–28.1)	43 (39.4–47.0)	25 (23.4–26.3)	32 (27.4–36.2)	27 (24.9–28.1)
Education	26 (25.0–26.8)	46 (42.8–49.0)	22 (20.9–23.3)	38 (33.4–42.8)	28 (25.6–29.5)
Health care	32 (31.3–33.1)	51 (48.5–52.9)	30 (28.7–30.9)	37 (31.8–41.7)	25 (22.4–26.7)
Accommodation and food	31 (29.4–32.8)	48 (43.2–51.9)	29 (27.1–31.5)	33 (27.6–39.2)	28 (24.5–30.9)
Public administration, arts	31 (29.8–31.5)	44 (41.8–46.8)	27 (25.3–27.8)	39 (35.9–42.2)	30 (29.0–32.0)
Health behaviors					
Sleep duration					
< 7 h	34 (33.6–34.9)	49 (47.2–50.7)	32 (31.0–33.0)	38 (35.6–39.9)	33 (31.8–33.8)
7 to 9 h	27 (26.9–27.7)	45 (43.2–46.0)	24 (23.7–24.9)	34 (32.5–35.7)	27 (26.8–28.0)
> 9 h	37 (35.1–38.9)	51 (46.4–55.7)	36 (32.8–38.5)	34 (28.3–39.0)	35 (32.0–38.4)
Smoking status					
Never	30 (29.4–30.5)	47 (45.7–48.5)	26 (25.8–27.2)	38 (36.1–40.2)	30 (28.2–29.8)
Current	32 (31.5–32.9)	53 (50.6–56.2)	29 (27.9–29.9)	39 (36.6–41.5)	33 (31.9–33.7)
Former	25 (24.5–25.8)	39 (36.2–41.2)	24 (23.3–25.3)	26 (23.9–27.9)	24 (22.9–25.0)
Alcohol consumption					
Never	34 (32.6–34.4)	47 (45.2–49.2)	31 (29.8–32.3)	35 (31.5–37.9)	30 (28.7–32.0)
Current	27 (26.6–27.5)	43 (41.2–45.0)	24 (23.0–24.3)	35 (32.8–36.6)	28 (27.2–28.5)
Former	36 (34.9–36.6)	50 (46.6–52.6)	35 (34.1–36.7)	36 (33.7–39.2)	34 (32.2–35.1)
Leisure-time physical activity					
Never/unable	36 (34.9–36.1)	48 (46.8–50.0)	34 (33.4–35.4)	36 (32.7–34.5)	34 (32.7–34.5)
Low	30 (29.2–30.5)	46 (43.8–47.6)	26 (25.6–27.2)	38 (35.6–40.4)	30 (29.3–31.2)
High	23 (22.7–23.9)	43 (40.6–45.1)	20 (19.2–20.8)	32 (29.3–34.2)	24 (23.0–24.7)
Clinical characteristics					
Hypertension (yes)	45 (44.5–45.8)	58 (56.2–59.4)	43 (42.2–44.5)	47 (45.2–49.2)	44 (42.6–44.6)
Diabetes (yes)	58 (56.8–59.4)	69 (67.0–72.0	58 (55.7–60.0)	56 (52.4–58.7)	56 (53.4–57.9)
Heart disease (yes)	36 (34.7–36.9)	55 (51.3–57.7)	33 (31.7–34.9)	43 (38.2–47.3)	35 (33.4–36.8)
Cancer (yes)	29 (28.1–30.5)	49 (44.2–52.8)	29 (27.0–30.2)	44 (36.2–52.3)	28 (25.5–29.8)
Health status					
Excellent/very good	22 (22.0–22.9)	36 (34.3–37.4)	19 (18.7–19.9)	30 (27.9–31.4)	23 (22.7–23.9)
Good	38 (37.7–39.0)	50 (48.2–51.9)	37 (36.0–38.0)	38 (36.0–40.6)	37 (36.4–38.3)
Fair/poor	46 (44.6–46.5)	60 (57.6–61.8)	45 (43.4–46.5)	43 (40.2–45.5)	43 (41.1–44.2)
Region of country					
Northeast	28 (27.2–29.0)	44 (40.3–46.9)	25 (23.7–26.4)	34 (31.2–37.1)	29 (27.7–30.3)
Midwest	31 (30.4–31.9)	45 (43.0–47.0)	29 (28.3–30.5)	37 (33.7–40.1)	31 (30.1–31.8)
South	31 (30.6–31.8)	48 (46.5–49.4)	27 (26.1–27.9)	35 (33.5–36.5)	30 (29.0–31.0)
West	26 (24.8–26.3)	43 (39.0–47.0)	24 (23.2–25.4)	34 (29.4–37.8)	25 (24.1–26.4)

Weighted estimates; the sample sizes are unweighted

### Obesity prevalence and trends by industry of employment

**Fig. 1:**
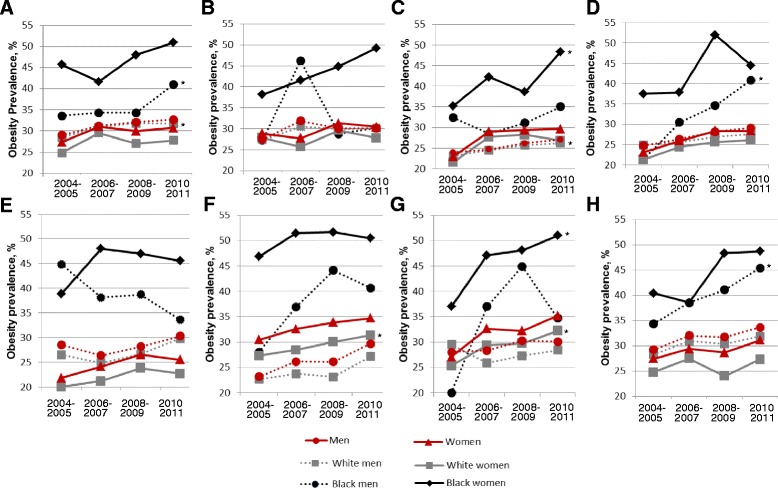
Trends in the Age-standardized Prevalence of Obesity by Industry of Employment for Men and Women and by Race-Gender Group, National Health Interview Survey, 2004–2011. **a** Manufacturing; Construction; Transportation; Wholesale trade; Agriculture; Utilities; Mining [WOMEN – (P for interaction: <0.001; P for trend = <0.001 for white women; P for trend=0.0795 for black women); MEN – (P for interaction: <0.001; P for trend <0.001 for white men; P for trend = 0.0089 for black men)] **b** Retail trade [WOMEN – (P for interaction: <0.001; P for trend = 0.3634 for white women; P for trend = 0.0591 for black women); MEN – (P for interaction: <0.001; P for trend = 0.3286 for white men; P for trend = 0.0468 for black men); MEN – (P for interaction: <0.001; P for trend = 0.3286 for white men; P for trend = 0.0468 for black men)] **c** Finance and insurance; Information; Real estate [WOMEN – (P for interaction: <0.001; P for trend = 0.0003 for white women; P for trend = 0.0267 for black women); MEN – (P for interaction: <0.001; P for trend = 0.3899 for white men; P for trend = 0.5209 for black men)] **d** Professional; Administrative; Management [WOMEN – (P for interaction: <0.001; P for trend=0.0326 for white women; P for trend=0.1386 for black women); MEN – P for interaction: <0.001; P for trend = 0.4876 for white men; P for trend = 0.0190 for black men)] **e** Education [WOMEN – (P for interaction: <0.001; P for trend = 0.0566 for white women; P for trend = 0.1279 for black women); MEN – (P for interaction: <0.001; P for trend = 0.3163 for white men; P for trend=0.4812 for black men)] **f** Health care and Social Assistance [WOMEN – (P for interaction: <0.001; P for trend = 0.0121 for white women; P for trend = 0.5412 for black women); MEN – (P for interaction: <0.001]; P for trend = 0.4356 for white men; P for trend = 0.0466 for black men)] **g** Accommodation and Food services [WOMEN – (P for interaction: <0.001; P for trend = 0.0721 for white women; P for trend = 0.1145 for black women); MEN – (P for interaction: <0.001]; P for trend = 0.7855 for white men; P for trend = 0.0209 for black men)] **h** Public Administration; Other services; Arts and Entertainment [WOMEN – (P for interaction: <0.001]; P for trend = 0.1832 for white women; P for trend = 0.0058 for black women); MEN – (P for interaction: <0.001]; P for trend = 0.2392 for white men; P for trend = 0.1360 for black men)] *obesity prevalence survey year 2004-2005 vs. 2010-2011 significantly different Direct standardization for age using the 2000 US Census as the standard population was employed

Participants employed in the healthcare industry had the highest overall age-standardized prevalence of obesity (32 %), while those in the education industry had the lowest (26 %). Figure 1 illustrates temporal trends from 2004 to 2011 for age-standardized prevalence of obesity by industry of employment overall and among black and white men and women. The prevalence of obesity appeared to increase for men and women over the study period across all employment industry categories. Women had a particularly high prevalence for health care and food/accommodation services, while men in the education sector experienced the greatest increase in obesity prevalence over time.

### Black-white differences in obesity by industry of employment

Table 2 presents age- and fully-adjusted prevalence ratios of obesity for blacks compared to whites by gender and industry of employment category. Compared to white women, black women had a significantly higher prevalence of obesity across all industries of employment (74 % excess prevalence in Education [95 % confidence interval (CI): 1.56, 1.93], 61 % [95 % CI: 1.46, 1.78] in Public Administration, 53 % [95 % CI: 1.33–1.77] in Food/Accommodation Services, 52 % [95 % CI: 1.39–1.67] in Manufacturing/Construction/Agriculture, 40 % [95 % CI: 1.24–1.57] in Retail, 37 % [95 % CI: 1.21–1.56] in Finance/Information, 44 % [95 % CI: 1.34–1.54] in Healthcare, and 33 % [95 % CI: 1.16, 1.52] in Professional/Management).

**Table 2:** Adjusted prevalence ratios of obesity for black compared to white women and men by industry of employment, National Health Interview Survey, 2004–2011 (*n* = 136,923)

	Model 1^a^: Age		Model 2^b^: Demographics		Model 3^c^: Occupational characteristics		Model 4^d^: Health Behaviors	
	PR	95 % CI	PR	95 % CI	PR	95 % CI	PR	95 % CI
	WOMEN							
Manufacturing/construction	1.72	(1.60–1.84)	1.66	(1.54–1.80)	1.61	(1.49–1.75)	1.52	(1.39–1.67)
Retail	1.55	(1.41–1.70)	1.49	(1.34–1.65)	1.48	(1.34–1.65)	1.40	(1.24–1.57)
Finances/information	1.60	(1.45–1.77)	1.55	(1.38–1.73)	1.53	(1.37–1.72)	1.37	(1.21–1.56)
Profess/admin/man	1.74	(1.57–1.93)	1.45	(1.28–1.64)	1.40	(1.23–1.59)	1.33	(1.16–1.52)
Education	2.05	(1.88–2.23)	1.87	(1.70–2.06)	1.85	(1.68–2.04)	1.74	(1.56–1.93)
Health care and social services	1.70	(1.62–1.80)	1.53	(1.44–1.63)	1.53	(1.43–1.62)	1.44	(1.34–1.54)
Accommodation and food	1.67	(1.50–1.87)	1.62	(1.44–1.84)	1.60	(1.42–1.82)	1.53	(1.33–1.77)
Public administration, arts	1.76	(1.63–1.90)	1.73	(1.59–1.89)	1.74	(1.59–1.89)	1.61	(1.46–1.78)
	PR	95 % CI	PR	95 % CI	PR	95 % CI	PR	95 % CI
	MEN							
Manufacturing/construction	1.18	(1.11–1.25)	1.23	(1.16–1.31)	1.22	(1.15–1.30)	1.19	(1.11–1.27)
Retail	1.14	(0.99–1.32)	1.16	(1.00–1.36)	1.16	(1.00–1.35)	1.09	(0.92–1.29)
Finances/information	1.27	(1.09–1.48)	1.22	(1.04–1.44)	1.21	(1.03–1.43)	1.18	(0.99–1.41)
Profess/admin/man	1.30	(1.14–1.47)	1.24	(1.08–1.42)	1.23	(1.07–1.41)	1.16	(0.99–1.35)
Education	1.39	(1.20–1.62)	1.42	(1.20–1.67)	1.40	(1.19–1.65)	1.39	(1.17–1.67)
Health care and social services	1.51	(1.30–1.77)	1.48	(1.24–1.76)	1.49	(1.25–1.77)	1.39	(1.15–1.69)
Accommodation and food	1.30	(1.08–1.56)	1.32	(1.08–1.62)	1.33	(1.09–1.63)	1.25	(0.99–1.58)
Public administration, arts	1.35	(1.23–1.48)	1.45	(1.32–1.60)	1.43	(1.30–1.58)	1.34	(1.20–1.49)

*PR* Prevalence Ratio, *CI* Confidence Interval

^a^Model 1 - adjusted for age categories

^b^Model 2 - adjusted Model 1 + gender, marital status, educational attainment, household income, living in poverty

^c^Model 3 - adjusted Model 2 + class of occupation, occupation

^d^Model 4 - adjusted Model 3 + smoking status, alcohol consumption, physical activity, sleep duration

Compared to white men, multivariable-adjusted obesity was more prevalent in black men employed in the following industries: Healthcare (PR = 1.39 [95 % CI: 1.15, 1.69]), Education (PR = 1.39 [95 % CI: 1.17, 1.67]), Public Administration (PR = 1.34 [95 % CI: 1.20, 1.49]), and Manufacturing/Construction/Agriculture (PR = 1.19 [95 % CI: 1.11, 1.27]). Obesity prevalence, however, was not different between black and white men in Retail, Finance/Information services, Professional/Management, and Food/Accommodation services industries. Demographics, occupational characteristics, and health behaviors did not substantially attenuate the obesity prevalence estimates for men and women. The supplemental table displays age-standardized prevalence of obesity by industry of employment among black and white men and women by each industry of employment without combining industries.

### Race-specific obesity prevalence trends by employment industry

Obesity prevalence significantly increased over the study period among black women in Finance/Information [35 % (28.2, 41.7) vs. 48 % (42.0, 55.0)] and Food/Accommodation services [37 % (29.4, 44.6) vs. 51 % (44.7, 57.2)] industries, while obesity prevalence significantly increased in Finance/Information [22 % (19.4, 23.7) vs. 27 % (24.1, 29.6)], Healthcare [27 % (25.7, 29.0) vs. 31 % (29.2, 33.5)], and Food/Accommodation services [25 % (21.9, 28.5) vs. 32 % (28.7, 35.9)] industries among white women (see Figure 1). Obesity prevalence was consistently higher among black women compared to white women over the study period in all industries.

Obesity prevalence significantly increased from 2004 to 2011 among black men in Manufacturing/Construction [34 % (30.3, 36.8) vs. 41 % (37.4, 44.5)], Professional/Management [22 % (15.1, 27.0) vs. 41 % (33.6, 48.1)], and Public Administration [34 % (28.4, 39.3) vs. 45 % (40.3, 51.5)] industries. Among white men, obesity prevalence significantly increased only in Manufacturing/Construction [28 % (27.3, 29.7) vs. 32 % (30.3, 33.1)]. The disparity in obesity appeared to decrease over time in Education while it appeared to increase over time in Professional management and Public Administration industries.

### Black-white differences in obesity by occupation within industry of employment

Table 3 shows the age-standardized prevalence of obesity by occupation within industry of employment overall and by race and gender. Obesity prevalence was higher among black men and women compared to their white counterparts in all occupations (except for men in support services occupations in Professional/Management services [26 vs. 27 %]). Obesity prevalence among black men and women widened with higher proportions of professionals within occupations for the Food/Accommodation services industry category. Furthermore, differences in obesity prevalence between blacks and whites for both women and men generally narrowed among laborers but increased among those in professional/management occupations.

**Table 3:** Age-standardized prevalence of obesity by occupation within industries overall and among 136,923 black and white men and women, National Health Interview Survey, 2004 to 2011

	Black women (*n* = 15,277)			White women (*n* = 58,784)			Black men (*n* = 10,023)			White men (*n* = 52,839)		
	No.^a^	%^b^	% Obese	No.^a^	%^b^	% Obese	No.^a^	%^b^	% Obese	No.^a^	%^b^	% Obese
All industries combined, *N* = 136,923												
Professional/management	1549	10	41	9316	16	23	1120	11	36	13,628	26	26
Support services	9195	62	46	38,435	66	26	2641	27	35	14,465	27	27
Laborers	4457	28	48	10,847	18	33	6196	62	37	24,521	47	32
Manufacturing/construction, *n* = 39,947												
Professional/management	151	8	45	1693	18	21	277	7	38	5331	22	28
Support services	591	29	44	3997	41	25	513	13	36	2992	13	30
Laborers	1403	63	48	4082	41	33	3417	80	36	15,359	65	32
Retail, *n* = 14,779												
Professional/management	(47)	(3)	(50)	343	5	25	(16)	(2)	(26)	385	7	24
Support services	1306	87	44	6213	86	28	525	62	32	3356	65	28
Laborers	150	10	40	670	9	33	307	36	35	1426	28	34
Finances/information, *n* = 12,769												
Professional/management	353	30	45	1878	30	24	171	28	32	1959	43	24
Support services	728	62	38	4265	67	27	253	42	30	1823	41	24
Laborers	103	8	42	217	3	39	224	30	34	736	17	29
Professional/administration/management, *n* = 12,709												
Professional/management	206	18	32	1971	40	21	242	24	36	3055	55	25
Support services	573	50	45	2453	49	26	166	16	26	837	16	27
Laborers	396	32	47	584	11	31	620	60	34	1539	29	29
Education, *n* = 13,858												
Professional/management	135	8	34	632	7	24	74	12	42	581	19	26
Support services	1212	71	43	6904	84	20	322	55	36	2057	64	25
Laborers	394	21	57	768	9	37	209	33	45	530	17	40
Health care and social services, *n* = 18,032												
Professional/management	277	6	40	998	9	25	67	10	35	396	18	25
Support services	3280	82	50	9532	86	29	382	62	37	1435	66	23
Laborers	525	12	58	622	5	44	179	29	37	339	16	31
Accommodation and food, *n* = 7682												
Professional/management	70	6	52	489	11	28	51	8	48	412	19	30
Support services	293	26	49	548	15	32	50	10	37	152	8	31
Laborers	818	68	47	2878	74	29	493	83	34	1407	73	26
Public administration/arts, *n* = 17,147												
Professional/management	360	16	43	1312	19	22	222	16	36	1509	23	28
Support services	1212	55	45	4523	66	26	430	30	39	1813	27	28
Laborers	668	29	42	1026	15	29	747	54	42	3185	49	34

Percentages are weighted estimates; the sample sizes are unweighted

^a^= Sample size for the entire race-gender subgroup; %

^b^= Percentage for the entire race-gender subgroup; () = estimates based on small sample size, which means less than 50 participants

In subsequent analyses, we found that employment status (employed vs. unemployed) in each industry remained similar or [should have been for] each racial group throughout the study period, and blacks were consistently more likely to be obese in each industry (see Additional file 1).

## Discussion

We found that the prevalence of obesity and obesity trends varied substantially by industries of employment and occupation overall as well as within and between race-gender groups. Participants employed in the healthcare industry had the highest overall age-standardized prevalence (30 %) while those in the education industry had the lowest (26 %). The prevalence of obesity appeared to increase for men and women over the study period across employment industry, and women had a particularly high prevalence for health care and food/accommodation services. Men in the education industry experienced the greatest increase in obesity prevalence over time.

Ironically, participants employed in the healthcare industry (32 %) had the highest overall age-standardized prevalence of obesity followed by food/accommodation services (31 %) and public administration (31 %). It is surprising that health care workers have the highest obesity rates because those in the healthcare industry are charged with advising the rest of the nation about healthy body weight recommendations. Of note, Bandura’s social learning theory suggests that patients are more likely to listen to health professionals if they serve as role models for the behaviors they tout or recommend. Our findings, along with those from Gu et. al., suggest the need to further study and intervene among workers in the healthcare industry given the overall high prevalence of obesity in this particular industry [43]. Furthermore, it should be noted that food industry workers often have to get up in the middle of the night to prepare food. These workers also work in, for instance, fast food chains that are open 24/7. Job conditions in both of these sectors may result in behaviors like extra snacking. The irregular sleep hours may also contribute to sleep deprivation, which has been shown to be associated with increased appetite and caloric intake.

As suggested by our study, along with previous investigations of overall obesity by employment industry category, complex aspects of the work environment that include inequitably distributed features likely influence population patterns of obesity [44–46]. For instance, both health care and food/accommodation services are known for requiring long and irregular working hours with inadequate rest/breaks and shift work; these factors are directly and indirectly associated with an excess of calories and metabolic/circadian deregulations.

Some hypothesized mechanisms by which the work environment could facilitate a differential prevalence of obesity include: 1) food environments in or around the workplace could enhance consumption of foods that increase obesity risk and vice versa; 2) physical environments or job requirements could increase the likelihood of sedentary activities during work hours; 3) job stress could affect lifestyle behaviors (e.g. alcohol drinking patterns, smoking, sedentary tendencies, sleep hygiene) related to obesity or weight gain; 4) coworker behaviors (e.g. eating habits; activity patterns) may negatively or positively influence the behaviors of other workers; 5) psychological job strain could modify endocrine factors associated with obesity; and 6) long work hours, shift work, and working overtime could lead to fatigue and promote behaviors (e.g. short sleep duration) that increase obesity risk while inhibiting behaviors associated with the prevention of weight gain and abdominal fat accumulation [21]. Short sleep, as a predictor of obesity, likely contributed, in part, to the racial/ethnic disparities in obesity trends we observed by industry of employment. In a previous study, we found that blacks were more likely to be short sleepers (<7 h within a 24-h period) than whites (37 vs. 28 %), that the black-white disparity was widest among professional occupations across industries of employment, and short sleep duration increased with increasing professional roles among blacks and decreased in whites [47–49]. Our cross-sectional approach to a mediation analysis of health behaviors, however, did not support the claim that health behaviors including sleep made a substantial contribution towards explaining overall racial differences in obesity by employment industry. Further research, perhaps by socioeconomic status within racial groups, is needed as black professionals, for instance, could experience unique factors (e.g. John Henryism or health-damaging work ethic to overcome adversity and succeed with limited resources; financial strain due to limited wealth; greater home stress) that contribute to obesity. Such findings could be masked when racial groups are analyzed as a monolith.

Black women had a significantly higher prevalence of obesity than white women across all industries of employment. Obesity prevalence was higher among black than white men in the Healthcare, Education, Public Administration, and Manufacturing sectors while non-significantly higher in most other industries. Men in the education industry experienced the greatest increase in obesity prevalence over time. These patterns in obesity among black men and women workers are consistent with other studies showing that blacks are also more likely than their white counterparts to report job stress and discrimination. Moreover, blacks tend to work more than one low-wage job and have positions with limited control over job demands in addition to being more likely to live in poverty despite employment [50–55].

Racial/ethnic disparities in obesity may be propagated through differential exposure to structural and informal workplace social hazards, which may create or exacerbate stressors that increase the likelihood of obesity. For instance, exposure to historical (e.g. overt racism) and contemporary forms (e.g. microaggressions, subtle indignities) of workplace discrimination and in broader society may contribute to psychosocial stress (e.g. depression, stereotype threat) as well as job strain or effort-reward imbalance [56–58]. Furthermore, shift work is more common in racial/ethnic minorities [59, 60], which may, at least partially, explain the racial differences in obesity by industry and occupation. Flatter inverse gradients between some socioeconomic status (SES) measures like occupation and common health-related outcomes among blacks compared to whites may also partially be explained by the higher obesity prevalence and subsequent disease risk among even blacks of higher SES [61]. It has been shown that blacks have more risk factors for comorbid conditions (e.g. obesity, hypertension, type 2 diabetes, sleep apnea) that may influence ones working conditions [62, 63]. Future research is needed in these areas.

Although future research is necessary, it is also possible that some industries could have become more or less biased towards hiring overweight individuals of any race over time. Even within the same industry, black workers and especially women, were more likely to be obese than their white counterparts, which generally remained consistent across the study period. Revealing a complex relationship between obesity and wages by race-gender group, Cawley et. al. found that overweight appears to lower the wages of white women while heavier black women as well as Hispanic women and men have lower wages that results from unobserved heterogeneity [64]. One potential explanation for the observation among white women was related to heavier white women being more likely to have a lower self-esteem than, for instance, black women. It is also possible that overweight white women face more discrimination in the workplace (e.g. getting hired, getting promoted) than other groups. Moreover, heavier black men had higher wages, which was apparently driven by underweight black men earning less compared to black men with healthy body weights rather than heavier black men earning more. Regarding the potential influence of occupations on the obesity epidemic and disparities, Lakdawalla and Philipson have hypothesized that technological changes have contributed to increased body weights by making lifestyles more sedentary. The authors also noted that technological influence varies by occupational class or socioeconomic status as well as race [65]. Furthermore, individuals with chronic health conditions (e.g. diabetes, cancer, heart disease) can influence occupational opportunities. For example, someone employed as a laborer might have a myocardial infarction and become a supermarket bagger since that job requires less strenuous physical activity. The health condition might, therefore, be a common prior cause of the cross-sectional occupation as well as change in BMI.

This study has limitations. First, we could not prospectively investigate employment industry and obesity risk among the employed due to the cross-sectional study design. We also relied on self-reported data. According to most studies, blacks and whites do not self-report their height and weight differently [66–71]; fewer studies have reported small differences [72–74]. Also, there was no access to data on diet/nutrition and medication use, which could influence obesity risk and differ by race. Furthermore, employment status was based on participants being employed during the week prior to the interview, and the impact of job security can be more variable for lower-SES and minority groups [75]. Studies investigating the influence of job security on obesity disparities would be useful. Furthermore, categories of occupation are broad and may include considerable heterogeneity. For instance, our categories likely correlate with wealth and some may include a disproportionate number of shift workers that we were unable to account for although shift work has been shown to differ by race and negatively impact obesity risk [45]. Lastly, we do not have residential data to account for the impact of racial residential segregation, which has been shown to influence health [76]. Importantly, Bleich et al. found no racial disparities in obesity among poor black and white women with a similar environmental context and income [77].

Despite the limitations, our study has important strengths and contributes to the public health literature. For instance, we had access to data on a large sample size overall and among black participants for which data is often scarce. The large sample size allowed for robust interaction testing along with racial, industry, and occupational stratification. Furthermore, we had eight consecutive years of industry data, which enhanced our power to investigate trends. Our data are also nationally representative and were recently collected. For easier interpretation of the data, we directly estimated prevalence ratios instead of odds ratios.

## Conclusions

Black-White differences in obesity varied by industry of employment and occupation, and have not appeared to change substantially over time. These findings demonstrate the need for further investigation of racial and sociocultural disparities in the work-obesity relationship to employ strategies designed to address disparities while improving health among all US workers. Interventions that prioritize workers who are employed in industries with high or increasing obesity are needed.

## Additional file

Additional file 1: Table S1. Age-Standardized Prevalence of Obesity by Industry of Employment among 136,923 US Black and White Men and Women, National Health Interview Survey, 2004–2011. (DOCX 14 kb)
