# Supplementary material for: Obesity trends by industry of employment in the United States, 2004 to 2011
Source: BMC Obes. 2016 Apr 2;3:20. doi: 10.1186/s40608-016-0100-x (PMC4818929; doi:10.1186/s40608-016-0100-x)
Supplement: Additional file 1: Table S1. — Age-Standardized Prevalence of Obesity by Industry of Employment among 136,923 US Black and White Men and Women, National Health Interview Survey, 2004–2011. (DOCX 14 kb) [file 40608_2016_100_MOESM1_ESM.docx]

|  | **Black women** | | **White women** | | **Black men** | | **White men** | |
| --- | --- | --- | --- | --- | --- | --- | --- | --- |
|  | **Sample size** | **% Obese (95% CI)** | **Sample size** | **% Obese (95% CI)** | **Sample size** | **% Obese (95% CI)** | **Sample size** | **% Obese (95% CI)** |
| Agriculture, Forestry, Fishing, and Hunting | 44 | 47.8  (29.5-66.1) | 488 | 27.5  (22.7-32.4) | 114 | 29.2  (16.4-41.9) | 1,361 | 29.5  (26.5-32.5) |
| Mining | 7 | 16.1  (9.2-41.3) | 90 | 22.6  (12.1-33.1) | 56 | 48.9  (33.7-64.0) | 576 | 32.6  (28.1-37.2) |
| Utilities | 57 | 53.1  (36.6-69.7) | 304 | 27.9  (22.1-33.8) | 130 | 33.8  (24.1-43.6) | 879 | 31.1  (27.5-34.8) |
| Construction | 87 | 43.3  (28.8-57.8) | 937 | 23.7  (20.2-27.1) | 857 | 32.6  (28.6-36.6) | 6,542 | 28.6  (27.3-30.0) |
| Manufacturing | 1,310 | 47.8  (44.3-51.3) | 5,715 | 29.6  (28.1-31.0) | 1,738 | 34.8  (31.9-37.7) | 9,163 | 31.1  (29.9-32.3) |
| Wholesale Trade | 150 | 48.2  (36.4-59.9) | 1,069 | 24.0  (20.7-27.3) | 286 | 37.4  (30.7-44.1) | 2,062 | 31.3  (28.7-33.9) |
| Retail Trade | 1,503 | 43.6  (39.7-47.5) | 7,240 | 27.8  (26.4-29.3) | 852 | 33.0  (27.8-38.2) | 5,184 | 29.3  (27.7-31.0) |
| Transportation and Warehousing | 503 | 46.8  (41.1-52.5) | 1,208 | 27.9  (24.9-30.9) | 1,046 | 37.2  (33.8-40.7) | 3,168 | 34.9  (32.9-36.8) |
| Information | 360 | 40.3  (32.7-47.8) | 1,633 | 28.9  (25.8-32.1) | 221 | 34.6  (24.5-44.8) | 1,514 | 26.2  (23.4-29.1) |
| Finance and Insurance | 657 | 41.4  (36.1-46.7) | 3,576 | 26.7  (24.8-28.6) | 219 | 33.4  (24.6-42.2) | 1,997 | 23.7  (21.2-26.2) |
| Real Estate and Rental and Leasing | 172 | 47.8  (37.5-58.1) | 1,183 | 22.7  (19.8-25.6) | 211 | 29.3  (21.9-36.8) | 1,026 | 27.0  (23.6-30.5) |
| Professional, Scientific, and Technical | 381 | 37.2  (30.2-44.1) | 3,299 | 21.3  (19.5-23.1) | 307 | 29.3  (20.3-38.3) | 3,524 | 24.0  (22.1-25.9) |
| Management of Companies and Enterprises | 4 | 41.5  (14.1-68.9) | 44 | 30.5  (13.8-47.2) | 1 | -- | 36 | 36.6  (17.4-55.9) |
| Administrative and Support and Waste Ma | 798 | 46.5  (41.7-51.3) | 1,689 | 32.0  (29.1-34.9) | 726 | 33.0  (27.5-38.4) | 1,900 | 31.8  (28.8-34.9) |
| Education Services | 1,751 | 45.9  (42.8-48.9) | 8,321 | 22.1  (20.9-23.3) | 610 | 38.1  (33.2-42.8) | 3,176 | 27.5  (25.6-29.5) |
| Health Care and Social Assistance | 4,052 | 50.7  (48.5-52.9) | 11,168 | 29.8  (28.7-30.9) | 634 | 36.8  (31.8-41.7) | 2,178 | 24.5  (22.4-26.7) |
| Arts, Entertainment, and Recreation | 200 | 41.4  (31.8-51.0) | 1,045 | 20.5  (17.4-23.7) | 236 | 33.5  (24.6-42.4) | 1,062 | 27.0  (23.4-30.7) |
| Accommodation and Food Services | 1,186 | 47.6  (43.2-51.9) | 3,921 | 29.3  (27.1-31.5) | 595 | 33.4  (27.6-39.2) | 1,980 | 27.7  (24.5-30.9) |
| Other Services (except Public Administration | 830 | 43.2  (38.7-47.7) | 2,879 | 25.9  (23.8-28.0) | 454 | 38.1  (32.5-43.6) | 2,275 | 29.7  (27.1-32.2) |
| Public Administration | 1,255 | 44.4  (40.9-47.8) | 2,975 | 29.3  (27.3-31.2) | 730 | 41.3  (37.0-45.5) | 3,236 | 32.1  (30.1-34.1) |

**Additional file 1: Supplemental Table. Age-Standardized Prevalence of Obesity by Industry of Employment among 136,923 US Black and White Men and Women, National Health Interview Survey, 2004-2011**

Weighted estimates; sample sizes are not weighted
